# Perforated Duodenal Diverticulum With Postoperative Diverticulum Bleeding Successfully Treated Using Transcatheter Arterial Embolization

**DOI:** 10.7759/cureus.18219

**Published:** 2021-09-23

**Authors:** Hidenori Tomida, Kan Nakagawa, Hideyasu Matsumura, Imai Shinichiro, Akimasa Matsushita, Shoichiro Koike

**Affiliations:** 1 Surgery, Shinshu University School of Medicine, Matsumoto, JPN; 2 Surgery, National Hospital Organization Matsumoto Medical Center, Matsumoto, JPN

**Keywords:** transcatheter arterial embolization, endoscopic hemostasis, duodenal diverticulum bleeding, duodenal diverticulum perforation, duodenal diverticulum

## Abstract

A diverticulum is a relatively common finding that is generally discovered incidentally; it is most commonly observed in the colon, followed by the duodenum. However, duodenal diverticulum perforation (DDP) is a rare complication. Due to its rarity, its diagnosis is often challenging and the appropriate treatment remains unclear, possibly contributing to its high mortality rate. Traditionally, surgical repair is the primary mode of treatment. However, with the recent advancements in medical technology, conservative management such as bowel rest and endoscopic drainage help successfully manage DDP. Duodenal diverticulum bleeding (DDB) is a rare cause of upper gastrointestinal bleeding. While endoscopic, angiographical, and surgical treatments have been performed to achieve hemostasis, there is no consensus regarding the optimal treatment for DDB. We describe a case of a perforated duodenal diverticulum (DD) with postoperative diverticulum bleeding. Our patient, an elderly female, complained of abdominal pain. Computed tomography images revealed free air in the retroperitoneum, and gastrointestinal perforation was suspected. During the emergency surgery, a perforated DD was detected in the third portion of the duodenum. Due to severe inflammation, diverticulectomy was not performed as it was deemed risky. Instead, we directly sutured the orifice using an omental patch. Duodenal leakage was observed from postoperative day (POD) 3 with bleeding from the remnant DD occurred on PODs 6 and 13. An attempt at endoscopic hemostasis failed, but transcatheter arterial embolization (TAE) was successfully performed. The postoperative course was complicated, and the patient died on POD 54. To the best of our knowledge, this is the first report on DD perforation with postoperative DDB. The remnant DD may be damaged by the digestive juices and result in bleeding. Precautionary measures for duodenal leakage should be undertaken when the DD is unresectable. Additionally, TAE is effective for postoperative DDB.

## Introduction

Following the colon, the duodenum is the second most common site of a diverticulum, and it is a relatively common finding that is generally discovered incidentally during endoscopic or imaging studies [1]. However, duodenal diverticulum perforation (DDP) is a rare complication. Due to its rarity, its diagnosis is often challenging and the appropriate treatment is still unclear, possibly contributing to its high mortality rate. A recent study reported an 8% mortality rate for DDP [2]. Traditionally, surgical repair is the primary mode of treatment. However, with the recent advancements in medical technology, conservative management such as bowel rest and endoscopic drainage have been successful in managing DDP [2]. Surgical treatments range from drainage-only to pancreatoduodenectomy (PD), but diverticulectomy (excision of the diverticulum and repair of the duodenum) is the most commonly performed intervention [2].

Duodenal diverticulum bleeding (DDB) is a rare cause of upper gastrointestinal (GI) bleeding, accounting for as low as 0.14% of all cases [3]. While endoscopic, angiographical, and surgical treatments have been performed to achieve hemostasis, there is no consensus regarding the optimal treatment for DDB [4]. Owing to the difficulty in reaching DD via standard upper GI endoscopy, very few cases of successful endoscopic hemostasis have been reported [5], and angiographic hemostasis should be considered as the first-line treatment for DDB [6].

## Case presentation

A frail 83-year-old female presented at our hospital with a five-day history of appetite loss and right-sided back pain. Her body weight, height, and body mass index were 33.0 kg, 150.0 cm, and 14.67, respectively. Her medical comorbidities included chronic heart failure, valvular disease, paroxysmal atrial fibrillation, and chronic kidney disease.

Blood examination results on admission revealed renal dysfunction (creatinine: 2.96 mg/dL), but the inflammatory marker levels were normal (white blood cell count: 4,260 U/L, C-reactive protein: 0.34 mg/dL). Hepatobiliary and pancreatic enzymes were within the normal range. Non-contrast computed tomography (CT) images revealed an old right rib fracture. The initial diagnoses were dehydration and rib fracture, and she was followed up as an inpatient and continuously received conservative treatment.

**Figure 1:**
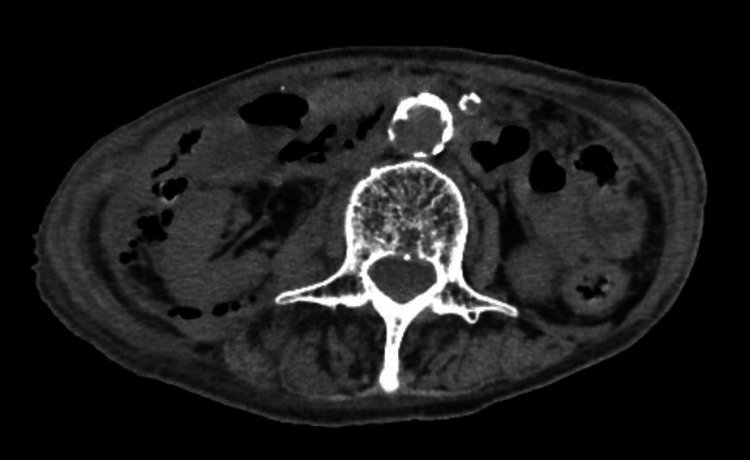
Computed tomography. Computed tomography (CT) scan showing retroperitoneal free air in the pararenal area.

On day 10 of hospitalization, the patient complained of right lower quadrant (RLQ) abdominal pain. Abdominal examination revealed tenderness in the RLQ without guarding. Blood test results revealed significantly elevated inflammatory marker levels (white blood cell count: 13,700 U/L, C-reactive protein: 28.73 mg/dL) and renal dysfunction (creatinine: 2.59 mg/dL). Non-contrast CT was repeated, and the images revealed the presence of retroperitoneal free air in the pararenal area (Figure 1). The CT results and the patient’s symptoms suggested gastrointestinal perforation, including duodenal perforation and colonic perforation.

**Figure 2:**
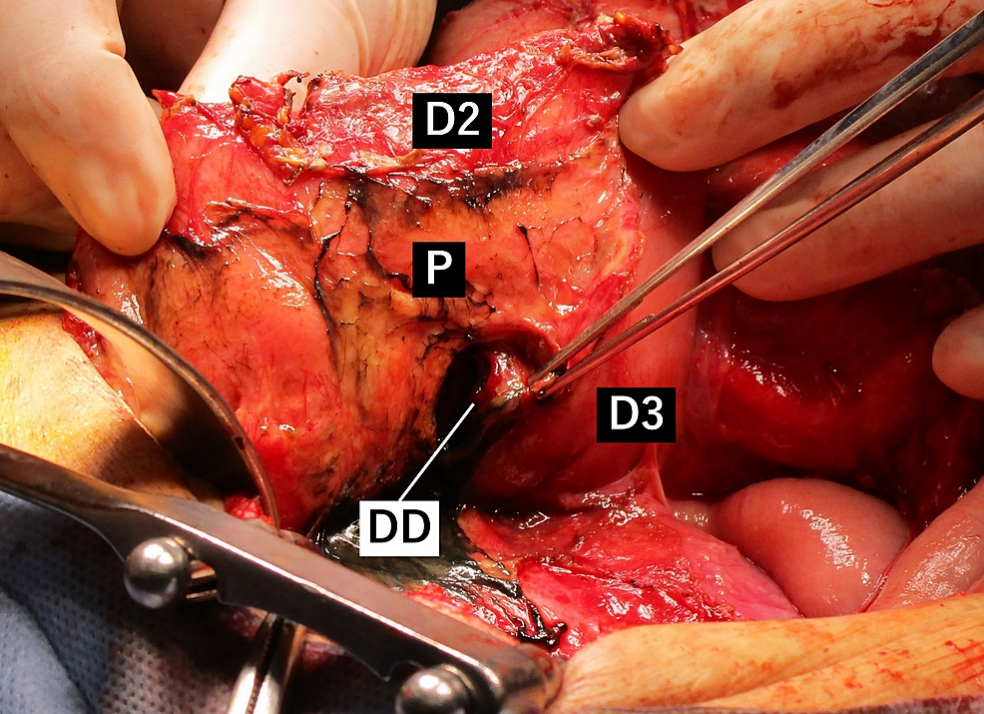
Intraoperative findings. Intraoperative photographs showing perforated diverticulum at the superior wall of D3. DD: duodenal diverticulum; D2: the second portion of duodenum; D3: the third portion of duodenum; P: pancreas.

We decided to perform an emergency operation. During exploratory laparotomy, a small amount of non-contaminated ascites was detected. The dorsal side of the ascending colon and the retroperitoneal space exhibited severe inflammatory changes. On the approach to the duodenum, periduodenal dark gray pus was observed. After mobilization of the duodenum, we discovered the perforated diverticulum at the superior wall of the third portion of the duodenum (Figure 2). There were no findings of mucosal inflammation suggestive of ulceration. Therefore, a diagnosis of a perforated DD in the third portion of the duodenum was made. The DD was encircled by the pancreatic head, and the entire diverticulum could not be visualized. Due to the severe damage of the surrounding tissue and the location of the DD, diverticulectomy was deemed risky. We instead directly sutured an omental patch to repair the orifice with subsequent placement of a retroperitoneal drain.

From postoperative day (POD) 3, leakage of bile and pancreatic juices continued (amylase and total bilirubin levels in the drainage fluid: 17,546 IU/L and 2.8 mg/dL, respectively), but this effectively controlled the inflammatory marker levels. We considered performing an endoscopic drainage of the bile juice, but the patient appeared unable to tolerate endoscopic retrograde cholangiopancreatography (ERCP) because of postoperative respiratory disorder.

**Figure 3:**
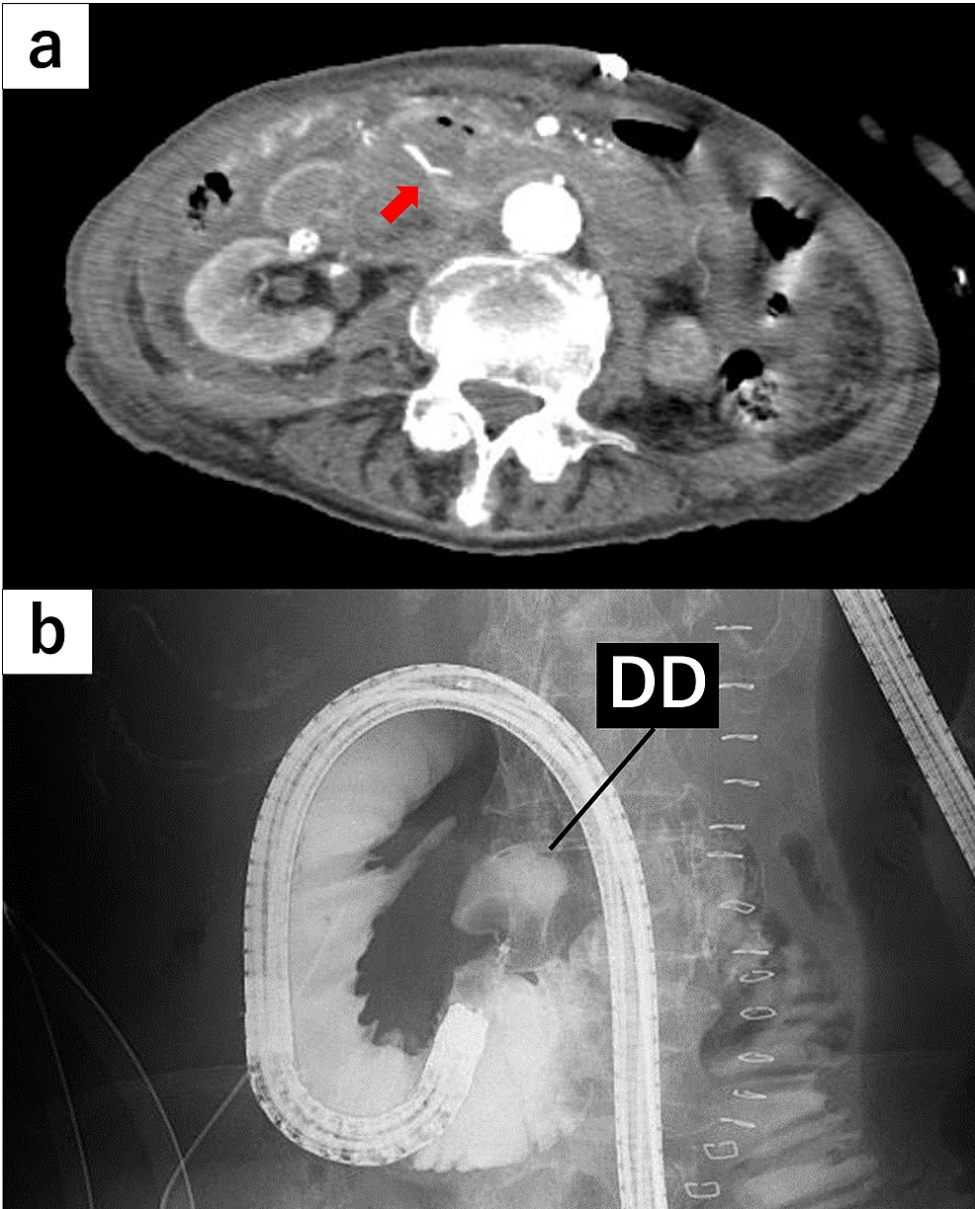
Computed tomography and duodenography. (a) CT scan showing extravasation (red arrow) from the neck of the duodenal diverticulum. (b) Duodenography showing the diverticulum with a small neck arising from the third portion of the duodenum. DD: duodenal diverticulum; CT: computed tomography

On POD 6, the drainage was observed to be bloody and contrast-enhanced CT images revealed extravasation from the neck of the DD (Figure 3a). Emergency endoscopy revealed acute bleeding from the eroded mucosa of the DD in the third portion of the duodenum. The exact source of bleeding could not be identified, but temporary hemostasis was successfully achieved using clips and injecting 5 cc of hypertonic saline-epinephrine solution. Intraprocedural duodenography revealed a DD with a small neck arising from the third portion of the duodenum (Figure 3b).

**Figure 4:**
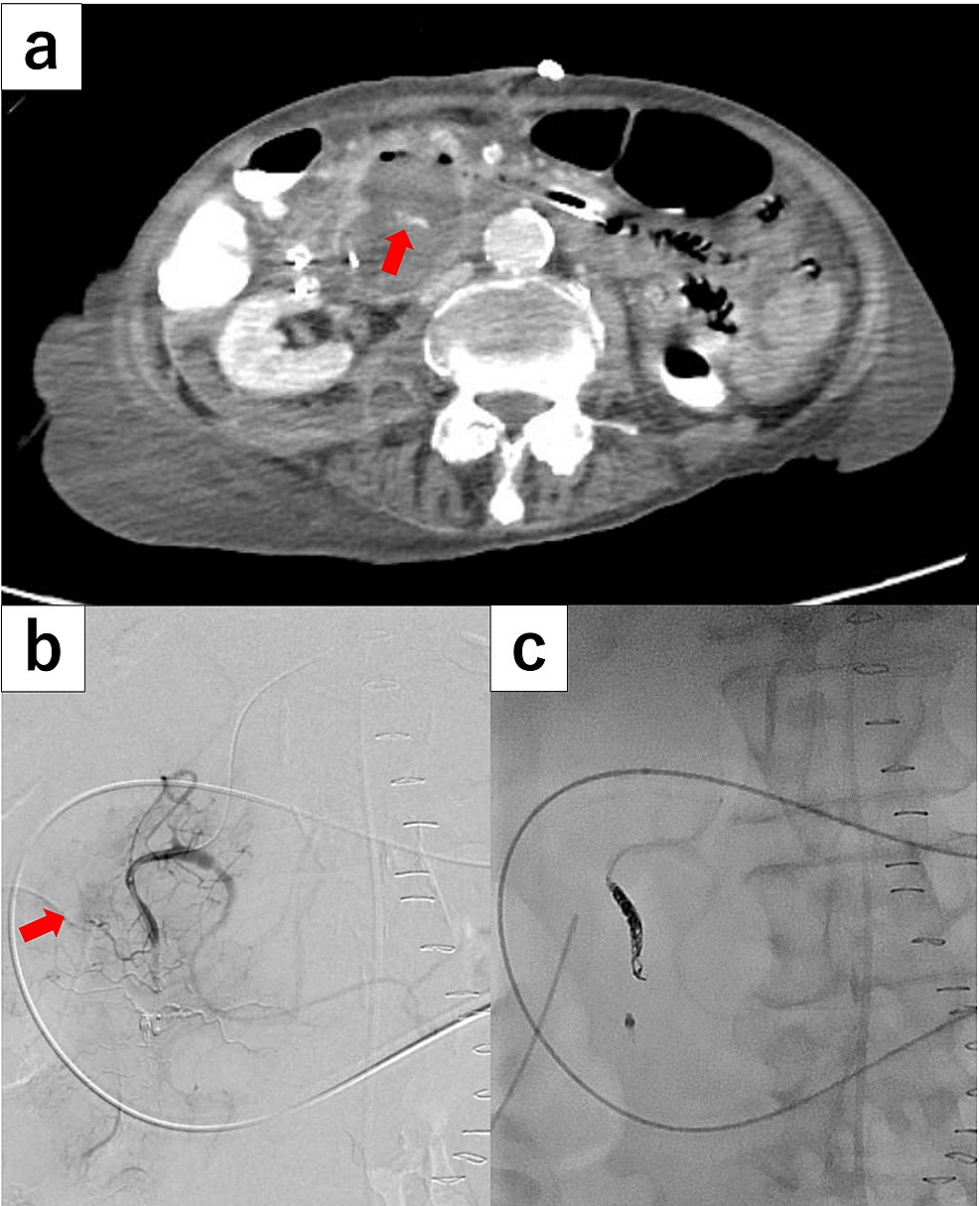
Computed tomography and emergency angiography. (a) CT scan showing extravasation (red arrow) from the neck of the duodenal diverticulum (as seen previously). (b) Emergency angiography showing extravasation (red arrow) from a distal branch of the anterior superior pancreaticoduodenal artery. (c) Hemostasis is achieved by embolization.

On POD 13, re-bleeding occurred due to the presence of bloody drainage. Contrast-enhanced CT images revealed extravasation from the same portion of the DD, and the previously attached clips were detached from the bleeding point (Figure 4a). Emergency angiography showed extravasation of the contrast material from a distal branch of the anterior superior pancreaticoduodenal artery, and subsequent hemostasis was achieved by embolization using eight microcoils (Figure 4b, c). After an endovascular procedure, no further bleeding was observed.

Complete wound dehiscence and a burst abdomen occurred on POD 26. Severe malnutrition was suspected as the cause, and re-closure was needed. While the patient was on the medical service, she was intubated and tracheotomy was performed for acute decompensation due to chronic heart failure. She subsequently developed multiorgan failure and died of acute kidney injury on POD 54.

## Discussion

A DD is a pouch attached to the duodenum and can be commonly found at the medial wall of the second (D2) or superior wall of the third (D3) portions of the duodenum [7]. It is a relatively common finding, and it is seen in up to 23% of asymptomatic cases, the majority of which remain asymptomatic throughout life [8]. However, complications such as inflammation, perforation, bleeding, pancreatitis, and obstructive jaundice can occur in 5%-10% of cases [1].

DDP is a rare but potentially severe complication that has been previously reported in the literature, mainly in case reports and series. The diagnosis of DDP is challenging and tends to be delayed because the symptoms are vague and non-specific. The duodenum is mainly located in the retroperitoneum, and the DD usually perforates into the retroperitoneal space. Therefore, the initial physical examination can be comparatively normal or indicate mild tenderness; some patients may complain of back pain, as in our case. CT provides an effective and accurate examination method in diagnosing DDP as it may reveal extraluminal gas and fluid collection [8]. Retroperitoneal free air in the pararenal area on CT images was reported as a suspicious finding for DDP [9].

Once DDP has been diagnosed, surgical management is traditionally performed. The operative method is selected based on the condition and location of the perforated DD and the general condition of the patient. A previous report revealed that diverticulectomy with drainage is the most commonly performed procedure [2]. Conservative treatment based on bowel rest and broad-spectrum antibiotics may be considered in some stable patients when an early diagnosis is made, or in elderly patients with severe comorbidities who are poor surgical candidates [10,11]. Although DDP is rare, the mortality rate was reportedly 13%-34% in older series [1,12]. A recent study of DDP cases from 1989 to 2012 indicated that the mortality rate was 8% [2].

Based on our search on PubMed (2013-present), there have been 29 reports of non-traumatic, non-iatrogenic DDP (Table 1) [11,13-32]. Including our case, the location of DDP was at D2 in 20 cases (67%) and D3 in six cases (20%). The most frequently performed treatments were diverticulectomy and direct suture in 15 cases (50%) and 14 cases (46%), respectively, similar to the previous report [2]. For the purpose of duodenal decompression, duodenostomy has been performed in three cases [13,28,32]. As a diversion procedure, duodenojejunostomy (two cases) and pyloric exclusion (one case) have been reported [16,19,25]. Biliary drainage was performed in three cases (bilio-duodenal drain, cystic duct tube, and postoperative endoscopic nasobiliary drainage, one case each) [14,28,29]. In some cases, dissection of the duodenum and performance of PD had to be undertaken because of severe inflammation and the risk of postoperative leakage [26, 28]. An intraduodenal suture using a Lap-Protector mini® was performed in one case in which DD was located near the inflamed pancreatic head and diverticulectomy would have been difficult [30]. Interestingly, three of the patients had undergone post-gastrectomy with Roux-en-Y gastric bypass reconstruction [28,32]. An increased intraduodenal pressure due to a blind loop has been a suggested cause of DDP [28]. A total of six cases (20%) were treated using conservative therapy, which consisted mainly of bowel rest, intravenous antibiotics, and endoscopic drainage. All cases had uncomplicated courses of recovery without recurrences of leakage or progression to a fatal condition. There were three deaths, which included our case, indicating a mortality rate of 10% (D2: one case and D3: two cases) [14,25].

**Table 1:** Cases of duodenal diverticulum perforation. ND: Not described.

Author	Year	Age (years)	Sex	Operation	Location	Complications
Barillaro et al. [13]	2013	83	F	Diverticulectomy, intraduodenally drainage tube	D3	None
Rossetti et al.[14]	2013	91	ND	Diverticulectomy, jejunostomy	D2	None
	2013	83	ND	Diverticulectomy, jejunostomy	D2	None
	2013	78	ND	Diverticulectomy	D2	Leakage
	2013	76	ND	No operation	D2	None
	2013	68	ND	Drainage	D2	Cardiac comorbidity/death
	2013	65	ND	Diverticulectomy	D2	None
	2013	48	ND	Diverticulectomy, jejunostomy, bilio-duodenal drain	D3	None
Haboubi et al. [15]	2014	78	F	Diverticulectomy	D2	None
Fujisaki et al. [16]	2014	69	M	Diverticulectomy, Roux-en Y duodenojejunostomy, cholecystectomy	D2	Leakage
Costa et al. [17]	2014	79	F	Partial duodenal resection, duodenojejunostomy	D4	None
Yeh [18]	2016	67	F	Laparoscopically diverticulectomy	D2	None
Glener et al. [19]	2016	65	F	Diverticulectomy, pyloric exclusion, gastrojejunostomy, jejunostomy	ND	None
Branco et al. [20]	2017	80	M	Diverticulectomy	D2	None
Fan and Talbot [21]	2017	75	F	No operation	D2	None
Degheili et al. [22]	2017	81	M	No operation	ND	None
Shirobe et al. [23]	2017	52	F	No operation	D2	None
Shen and Leong [24]	2017	85	M	Partial duodenal resection, duodenojejunostomy	ND	ND
Khan et al. [25]	2018	82	F	Diverticulectomy, duodenojejunostomy, gastrostomy	D3	Sepsis/death
Kim and Park [11]	2018	68	M	No operation	ND	None
Philip and Cocieru [26]	2019	70	F	Pancreatoduodenectomy	D2	None
Sahned et al. [27]	2019	77	F	Diverticulectomy, jejunal serosal patch	D2	None
Yagi et al. [28]	2019	66	M	Pancreatoduodenectomy	D2	None
	2019	52	M	Direct suture, duodenostomy, jejunostomy, cystic duct tube	D2 and D3	None
Shimada et al. [29]	2020	60	F	Omental patch	D2	Leakage
Maki et al. [30]	2020	94	F	Intraduodenal diverticulectomy	D2	None
Moysidis et al. [31]	2020	51	F	Diverticulectomy	D2	None
	2020	58	F	No operation	D2	Upper respiratory infection
Kabelitz et al. [32]	2020	47	F	Direct suture, duodenostomy	D3	None
Our case	2021	83	F	Direct suture, omental patch	D3	Leakage, Diverticulum bleeding/death

Except for our case, there have been no reported cases of DDP with postoperative DDB. In our case, the unresectable portion of the DD may have been damaged by postoperative leakage, resulting in DDB. Pancreatic juice is activated by bile juice and duodenal juice. Activated pancreatic juice can cause severe damage to fragile tissues, and bleeding can occur from eroded vessels adjacent to the inflamed DD. Based on the above, postoperative duodenal leakage may be a potential risk factor for DDB, and drainage of digestive juices (duodenal, bile, and pancreatic juice) should be considered. The effectiveness of endoscopic nasobiliary and nasopancreatic drainage for postoperative leakage of DDP has been reported [29]. However, postoperative ERCP for unstable patients, as in our case, presents several risks. In cases wherein postoperative leakage is highly suspected, intraoperative procedures for drainage of bile or duodenal juice, such as cystic duct tube placement and duodenostomy, should be considered.

DDB is extremely rare and accounts for as low as 0.14% of all upper GI bleeding [3]. Bleeding can occur from the eroded vessels adjacent to the inflamed diverticulum [33]. DDB has been reported to occur most commonly from the D3 and fourth portions (D4) but rarely from D2 [34]. Gastrointestinal endoscopy is usually the initial examination for GI bleeding, which also allows for hemostasis if bleeding is detected. However, performing endoscopic examination in the distal duodenal lumen is challenging due to poor operability. As it is difficult to access the DD via a standard upper GI endoscopy, very few cases of successful endoscopic hemostasis have been reported to date [5]. Transcatheter arterial embolization (TAE) has been performed as a diagnostic and therapeutic procedure for GI bleeding. Several studies have reported that an angiographic embolization is considered an initial therapy for colonic bleeding [35,36]. TAE for upper GI bleeding had been used when endoscopic hemostasis was unsuccessful and surgical intervention is not recommended [37]. TAE is less invasive than surgery and is a viable option for candidates with a poor medical condition, such as for elderly patients and surgically high-risk cases [37]. It is also believed that performing endoscopic hemostasis for bleeding from D3 or D4 is sometimes difficult due to their positions; therefore, TAE and surgical intervention should be considered in these portions [38].

Including our case, a summary of all 11 cases (2005-present) is provided in Table 2 [5,6,39-45]. The bleeding site was D2 in five cases and D3 in five cases. In nine cases, endoscopic treatment was initially performed, but five cases required further endoscopic, radiological, or surgical treatment. A notable fact is that in three of the five cases of endoscopic failure, TAE achieved hemostasis.

**Table 2:** Cases of duodenal diverticulum bleeding. TAE: transcatheter arterial embolization; D2: second portion of the duodenum; D3: third portion of the duodenum; D4: fourth portion of the duodenum.

Author	Year	Age (years)	Sex	Location	Treatment		
					1st	2nd	3rd
Kua et al. [39]	2005	69	F	D2	Endoscopic	surgery	
Kobayashi et al. [40]	2008	59	M	D2	Endoscopic	TAE	
Kwon et al. [6]	2009	69	F	D3	TAE	surgery	
Lee et al. [41]	2010	67	M	D2	Endoscopic	TAE	Endoscopic
Nishiyama et al. [42]	2012	71	F	D2	Endoscopic		
Whilhelmsen et al. [43]	2014	69	M	D3	Endoscopic	TAE	
Matuso et al. [5]	2015	68	F	D3	Endoscopic		
Hatt and Mills [44]	2016	66	F	D4	Surgery		
Chatila et al. [45]	2019	57	M	D2	Endoscopic		
	2019	79	F	D3	Endoscopic		
Our case	2021	83	F	D3	Endoscopic	TAE	

## Conclusions

DD is a relatively common finding and is rarely complicated; however, DDP is a potentially serious complication. Management of DDP is still controversial, but the remnant DD could be damaged by postoperative leakage, which could result in DDB. During surgery, precautionary measures for duodenal leakage are important when the DD is unresectable. TAE for DDB may be as effective as endoscopic hemostasis, including postoperative cases. When endoscopy fails to reveal the exact source of bleeding, TAE should be investigated.
